# 306. Development a Predictive Score for Severe Coronavirus Disease 2019 (COVID-19) from High Risk Factor of Patient Described by Thai Government Policy

**DOI:** 10.1093/ofid/ofac492.384

**Published:** 2022-12-15

**Authors:** Preudtipong Noopetch, Rutporn Benchamanon, Samerjan Kongsuwan, Chatree Chai-adisaksopha

**Affiliations:** Internal medicine, Hatyai hospital, Hatyai, Songkhla, Thailand; Obstetric and Gynecology department, Hatyai hospital, Hatyai, Songkhla, Thailand; Internal medicine department, Hatyai hospital, Hatyai, Songkhla, Thailand; Chiang Mai University, Amphoe muang, Chiang Mai, Thailand

## Abstract

**Background:**

Thai government had a policy defined population groups that are at risk of severe COVID-19 infection. It is called “608 group” consisting of age more than 60 years old, obesity, diabetes mellitus, cancer, cerebrovascular disease, respiratory disease, chronic kidney disease, HIV infection, and pregnancy. But no study has evaluated performance of this policy. We aimed to develop parameter risk-based scoring system from Thai policy for diagnosis of severe COVID-19 infection.

**Methods:**

A study was carried out in 11,677 patients with confirmed COVID-19 infection were admitted to Hatyai hospital, Songkhla, Thailand from 1 April 2021 to 31 December 2021. Patients were categorized to severe COVID-19 infection if their oxygen saturation less than 94% or need oxygen supplement. Multivariable logistic regression was used to explore for predictors. The logistic coefficients were transformed to risk-based scoring system.

**Results:**

A total of 11,677 patients were included in analysis and predictive model development, 1036 (8.88%) patients were severe COVID-19 infection, and 10,631 (91.12%) patients were non-severe COVID-19 infection. Age more than 60 years old, obesity, diabetes, cancer, cerebrovascular disease, respiratory disease, chronic kidney disease, HIV infection, and pregnancy were used for derivation of the scoring system. The score-based model showed area under ROC of 0.81 (95%CI 0.79 – 0.82). The scoring system ranged from 0 to 40 was classified into 3 subcategories for clinical practicability. The sensitivity and specificity for predictive of severe COVID-19 were 81.18% and 69.83% for low risk patient, 70.56% and 80.79% in moderate risk patient, and 54.92% and 89.81% in high risk patient.

**Figure:**
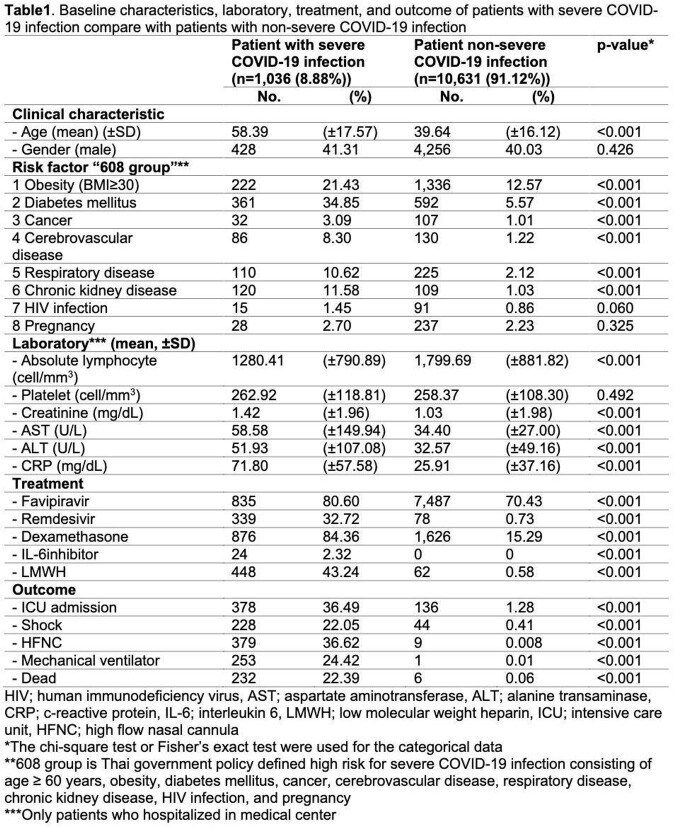

**Figure:**
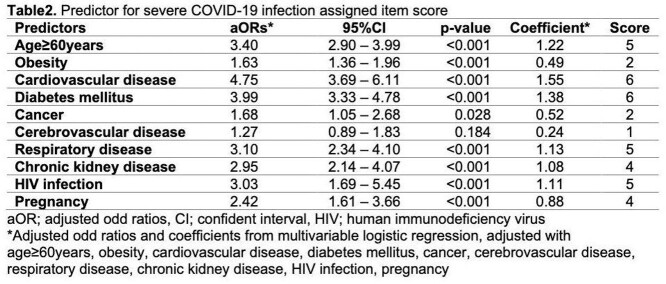

**Figure:**
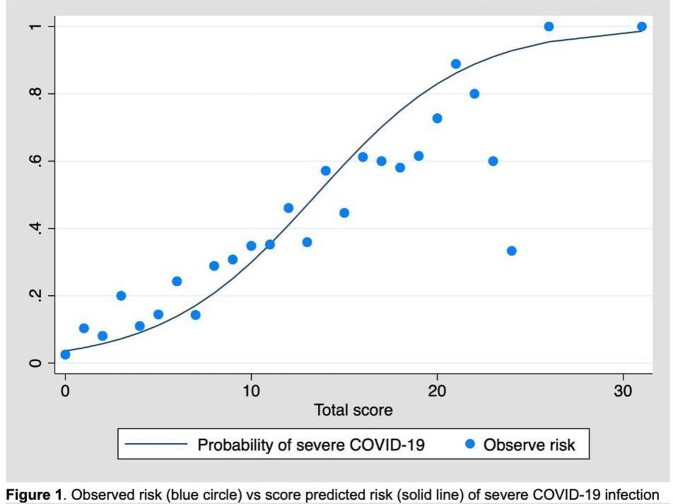

**Conclusion:**

This simplified risk-based scoring system for prediction severe COVID-19 disease could aid general physicians or internist in evaluation and triage of patients who present with COVID-19 infection and help physicians in management and prioritization of patients in outbreak situation.

**Figure:**
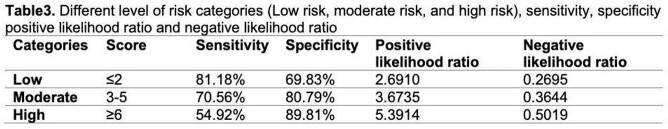

**Figure:**
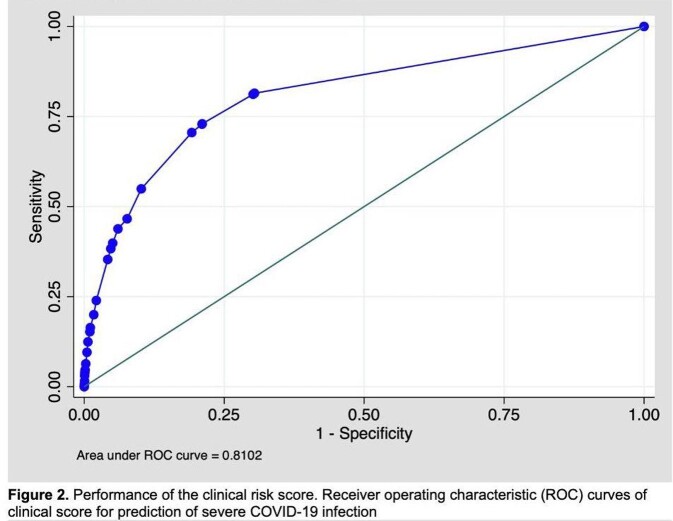

**Disclosures:**

**All Authors**: No reported disclosures.

